# The dietary inflammation index and body mass index mediate the association between the dietary index for gut microbiota and sarcopenia in older women: evidence from NHANES 2011–2018

**DOI:** 10.3389/fnut.2025.1624844

**Published:** 2025-08-18

**Authors:** Xiaoyan Zhang, Liangzhi Wu, Yutian He, Shuyao Zhang, Wenfeng Hua

**Affiliations:** ^1^Department of Pharmacy, Guangzhou Red Cross Hospital (Guangzhou Red Cross Hospital of Jinan University), Guangzhou, China; ^2^Department of Gynecology, The Affiliated Guangdong Second Provincial General Hospital of Jinan University, Guangzhou, China; ^3^Research Institute for Maternal and Child Health, The Affiliated Guangdong Second Provincial General Hospital of Jinan University, Guangzhou, China

**Keywords:** DI-GM, dysbiosis, gut microbiota, sarcopenia, NHANES

## Abstract

**Background:**

The dietary index of gut microbiota (DI-GM) was developed to assess dietary quality by reflecting the diversity of the gut microbiota composition. This study examined the relationship between DI-GM and sarcopenia risk and evaluated the potential moderating effects of diet-related factors on sarcopenia risk in adult individuals.

**Methods:**

This cross-sectional study included 8,872 adults from the National Health and Nutrition Examination Survey (NHANES) database between 2011 and 2018. Weighted multivariable logistic regression, restricted cubic splines (RCS), and subgroup analyses were used to investigate the association between DI-GM and sarcopenia. Mediation analysis was employed to examine the impact of the dietary inflammatory index (DII), systemic immune-inflammatory index (SII), and body mass index (BMI) on the relationship between DI-GM and sarcopenia.

**Results:**

Among the eligible participants, 773 (8.71%) had sarcopenia. The mean DI-GM value was significantly lower in the sarcopenia group than in the non-sarcopenia group (4.76 vs. 4.99, *p* < 0.001). Multivariable logistic regression analysis revealed a negative association between DI-GM and the risk of sarcopenia, irrespective of whether the independent variable was analyzed as a continuous variable or in quartiles in the fully adjusted model (Model 3, continuous variable: OR = 0.91, 95% confidence interval (CI): 0.88–0.95, *p* < 0.001; Q4 vs. Q1: OR = 0.67, 95% CI = 0.56–0.80, *p* < 0.001, *p* for trend<0.001). The RCS curves illustrated a non-linear relationship between DI-GM and sarcopenia risk. Subgroup analyses indicated an inverse relationship between DI-GM and sarcopenia risk across various covariates in the study. Mediation analysis demonstrated that 46.32, 2.29, and 29.63% of the association between DI-GM and sarcopenia was mediated by the DII, SII, and BMI, respectively.

**Conclusion:**

A lower DI-GM score was strongly associated with an increased risk of sarcopenia, particularly among older adults, women, individuals with hypertension, and individuals with reduced physical activity. The DII and BMI may mediate the relationship between DI-GM and sarcopenia risk, suggesting that a diet promoting gut health could be an effective strategy for preventing sarcopenia. Additional longitudinal or interventional studies are required to substantiate the findings of this study.

## Introduction

Sarcopenia is a progressive condition that affects the skeletal muscles and is characterized by a decline in muscle mass and function (1). This condition is associated with a heightened risk of falls, reduced functionality, frailty, and mortality. It is estimated that sarcopenia affects between 10 and 16% of the global elderly population. It generally arises with age and is shaped by hereditary, environmental, and lifestyle factors. Factors such as smoking, physical inactivity, malnutrition, inadequate sleep, and diabetes adversely affect muscle health (1–3). Resistance training is the primary treatment for sarcopenia; however, its effectiveness is often hindered by low patient adherence. Numerous studies have explored biomarkers, dietary interventions, and medications to enhance the effects of resistance training (4–6). The link between muscle health and dietary habits suggests that dietary modification and nutritional supplements could be promising intervention strategies.

A common condition among the elderly, known as inflammaging, is characterized by increased inflammatory markers in the bloodstream (7). This condition increases the risk of chronic diseases, disability, frailty, and early mortality. Inflammageing also increases the likelihood of various health issues, including cancer, diabetes, dementia, depression, and sarcopenia (7–9). Studies have found that the gut microbiota can affect muscle mass and function through several mechanisms, including inflammation, immune response, metabolism, endocrine activity, and insulin sensitivity (10, 11). Additionally, age-related decline in intestinal barrier function allows live bacteria and their byproducts to enter the bloodstream, potentially causing persistent low-grade inflammation, even in the absence of detectable pathogens (12). There is substantial evidence supporting the potential of targeting the gut-muscle axis to alleviate the traits associated with sarcopenia. Zhang et al. (13) demonstrated that *Bifidobacterium animalis* subsp. *lactis* Probio-M8 improved the physical performance of patients with sarcopenia and enriched beneficial metabolites, thereby enhancing the health of older adults. Similarly, Chen et al. (14) showed that *Lactobacillus casei* Shirota attenuates inflammaging through the gut-muscle axis in mice with age-related muscle impairment. These findings suggest that dietary interventions targeting the gut-muscle axis may help reduce the risk of sarcopenia.

Accumulating evidence indicates that dietary patterns substantially influence gut microbiota composition and have been implicated in the etiology of sarcopenia. Recent studies have indicated that high adherence to the Mediterranean diet is correlated with enhanced muscle strength and function and a decreased risk of sarcopenia (15–17). This dietary pattern prioritizes the consumption of fruits, vegetables, legumes, whole grains, nuts, and olive oil as essential components of the daily diet. The muscle-protective effects of the Mediterranean diet are attributed to its balanced vitamins and phytochemicals with antioxidant properties, which modulate the intestinal microbiome, favoring bacteria involved in synthesizing bioactive compounds, such as short-chain fatty acids, which counteract inflammation, anabolic resistance, and tissue degeneration (18–20). Conversely, increased consumption of unhealthy foods, characteristic of a Western diet, which is deficient in fruits, vegetables, whole grains, fish, nuts, and seeds, has been associated with a higher prevalence of sarcopenia. This is attributed to the promotion of harmful gut bacteria that contribute to inflammation and disease development by compromising the immune system (21–23). Furthermore, a Western diet is frequently associated with obesity and insulin resistance, which can modify the composition and diversity of the gut microbiota, ultimately influencing skeletal muscle metabolism and functionality (23–25).

Dietary patterns significantly influence the composition of the gut microbiota, underscoring the importance of employing dietary indices to elucidate the relationship between the gut microbiome and disease risk (26, 27). Recently, Kase et al. (28) developed a novel dietary index for the gut microbiota (DI-GM) to reflect the gut microbiota diversity and abundance according to dietary quality. This index assesses the impact of diet on the gut microbiota through 14 components identified as either beneficial or detrimental to gut health, thereby effectively capturing the relationship between dietary quality and gut microbiota diversity.

Recent studies have indicated that an elevated DI-GM score correlates with a reduced risk of chronic kidney disease, diabetes, non-alcoholic fatty liver disease, stroke, frailty, and aging (29–34). These conditions are closely associated with sarcopenia. However, the interrelationship between DI-GM, inflammaging, and sarcopenia remains unclear. This cross-sectional study aimed to fill this knowledge gap by analyzing the association between DI-GM and sarcopenia using NHANES data. Additionally, this study provides valuable insights for developing targeted dietary interventions to alleviate sarcopenia.

## Methods

### Data source

This study utilized data from the 2011–2018 NHANES, a comprehensive cross-sectional survey conducted every 2 years. This survey gathered information on dietary habits, nutritional status, health conditions, and lifestyle behaviors of the non-institutionalized U.S. population using a multistage probability-sampling method. These data are publicly accessible from the National Center for Health Statistics (NCHS), a division of the Centers for Disease Control and Prevention (CDC). The NHANES protocols received approval from the NCHS Ethics Review Board, and all participants provided written informed consent. Further details can be found at http://www.cdc.gov/nchs/nhanes/index.htm.

### Study design and population

**Figure 1:**
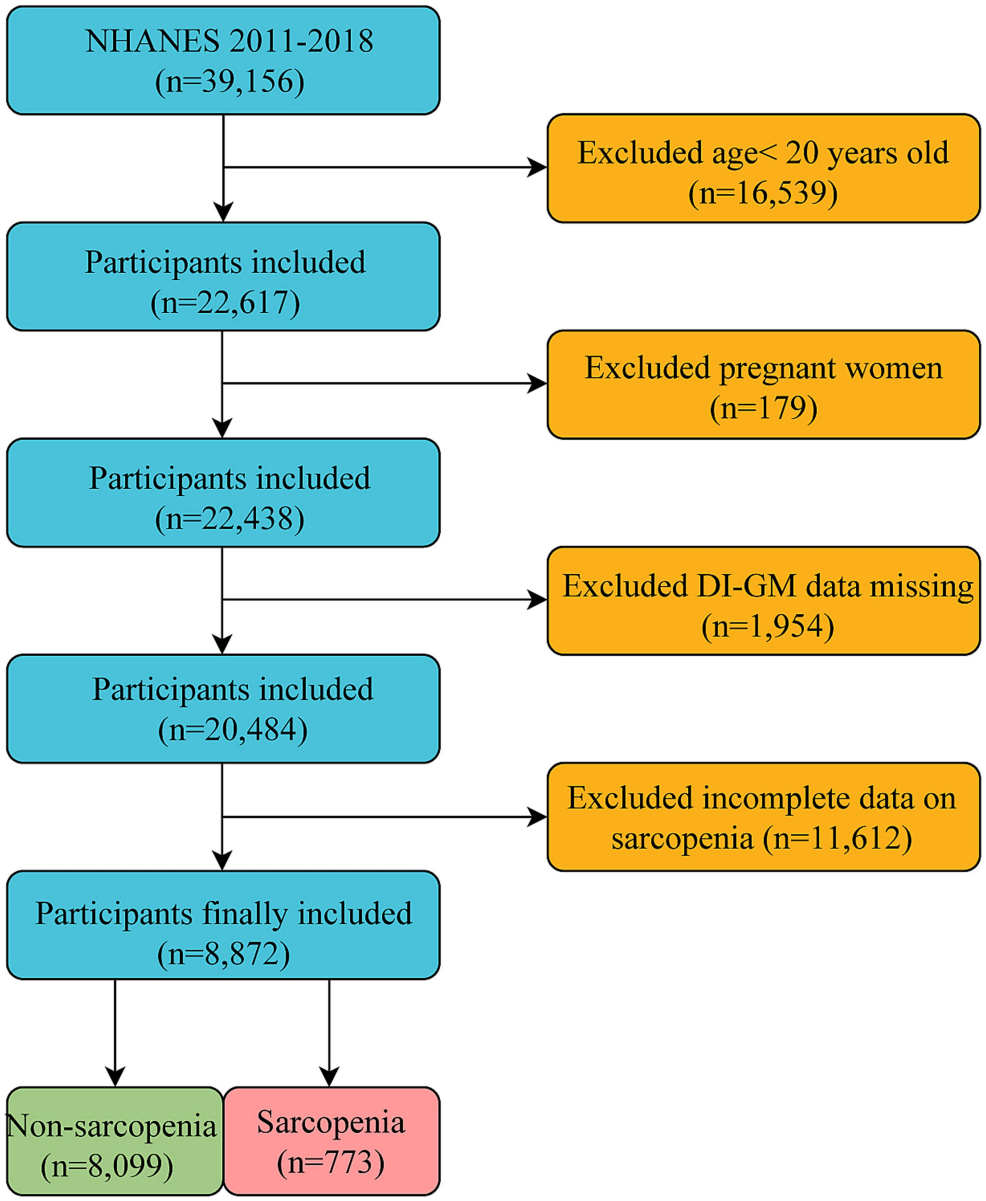
Flow chart for the inclusion and exclusion of study participants.

Our study focused on adults aged ≥20 years who were not pregnant, representing the non-institutionalized civilian population of the United States. We obtained a comprehensive dataset that included DI-GM components and sarcopenia status from an initial cohort of 39,156 participants. Several groups were excluded from the study: those aged <20 years (*n* = 16,539), pregnant women (*n* = 179), individuals with missing DI-GM components (*n* = 1,954), and those without sarcopenia information (*n* = 11,612). The final analysis sample comprised 8,872 eligible participants (Figure 1).

### Calculation of DI-GM

This study used the scoring system proposed by Kase et al. (28) to calculate the DI-GM using 14 foods and nutrients. The DI-GM includes 10 beneficial components (avocado, broccoli, chickpeas, coffee, cranberries, fermented dairy, fiber, green tea, soy, and whole grains) and four unfavorable components (red meat, processed meat, refined grains, and high-fat foods). We averaged the results from two 24-h dietary recall interviews per participant to determine the DI-GM scores. Participants with only one reliable dietary recall were excluded (28). A score of 1 indicated consumption above the median for beneficial components and below the median for detrimental components, whereas 0 indicated the opposite. The total score ranged from 0 to 13, with higher scores suggesting a healthier gut microbiota. Based on previous studies (32, 35, 36), participants were categorized into four groups: 0–3, 4, 5, and ≥6.

### Definition of sarcopenia

Appendicular skeletal muscle mass (ASM) was assessed using dual-energy X-ray absorptiometry in the NHANES database. The sarcopenia index (SI) was calculated using the following formula: SI = total ASM (kg)/BMI (kg/m^2^). Sarcopenia was defined as an SI of less than 0.789 in men and less than 0.521 in women, according to the criteria established by the Foundation for the National Institutes of Health Biomarkers Consortium (37).

### Calculation of DII and SII

The dietary inflammatory index (DII) was calculated using a previously established formula to assess the inflammatory potential of the diet across different populations (38). Individual food component intakes were aligned with the global mean intake, and a *z*-score was computed by comparing the reported intake to the global mean divided by the standard deviation. The *z*-score was then converted into a standardized percentile and adjusted for the inflammatory impact of each food parameter, as in previous studies (39). The DII scores for the food parameters were combined to derive an overall DII score, with higher scores indicating a more proinflammatory diet. The systemic immune-inflammation index (SII) was calculated using the formula from a previous study, specifically SII = P × N/L, where P, N, and L represent platelet, neutrophil, and lymphocyte counts, respectively, expressed as ×10^3^ cells/mL (40). In this study, both the DII and SII were considered as mediating variables.

### Covariates

This study investigated a variety of factors, including demographic characteristics such as sex, age, race, marital status, education level, and poverty income ratio (PIR); lifestyle habits such as alcohol consumption and smoking; health conditions including cardiovascular disease (CVD), diabetes, and hypertension; daily energy intake (kcal/day); and physical activity levels, categorized as low physical activity (<500 metabolic equivalent (MET) scores/week) and high physical activity (≥500 MET/week) (41). Comprehensive information on the methodologies employed to collect data on these variables is available on the official NHANES website.^1^

### Statistical analysis

The surveys employed a multistage clustered design, and the statistical analyses followed the National Health and NHANES sampling weights as recommended by the CDC. We compared the baseline characteristics of the participants using descriptive analyses based on sarcopenia status and DI-GM quartiles. Continuous variables are presented as means with standard errors (SEs), and categorical variables are presented as counts and percentages (%). To evaluate the baseline characteristics, we used weighted linear regression and chi-square tests. To address the issue of missing data, we utilized multivariate multiple imputations (MI), employing five replications and chained equations within the R MI procedure to effectively account for the missing data. We utilized weighted multivariate logistic regression to examine the association between DI-GM and sarcopenia risk, while accounting for potential confounding variables. Subgroup analyses were conducted to explore this association across demographic and clinical groups, considering factors such as sex, age, BMI, PIR, CVD, smoking, drinking, physical activity, daily energy intake, and the presence of hypertension and diabetes mellitus. We employed restricted cubic spline (RCS) curves and threshold effect analysis to investigate the potential non-linear relationship between DI-GM scores and sarcopenia risk. Finally, a mediation analysis was performed to determine whether the effect of DI-GM on the risk of sarcopenia was mediated by DII, SII, and BMI. Statistical analyses were conducted using R (version 4.4.0) and Zstats (version 1.0) software, with statistical significance set at *p* < 0.05.

## Results

### Participant characteristics

As shown in Table 1, of the 8,872 eligible participants, 773 had sarcopenia. Those in the sarcopenia group were generally older and had a higher BMI, lower income, reduced daily energy intake, and lower education level. They also showed a higher prevalence of being married, Mexican American, abstaining from alcohol, engaging in low physical activity, and having conditions such as CVD, diabetes, and hypertension than those in the non-sarcopenia group. Moreover, the average DI-GM value was significantly lower in the sarcopenia group than in the non-sarcopenia group (4.76 vs. 4.99, *p* < 0.001). Participants with lower DI-GM scores also had higher dietary inflammatory index (DII) and systemic immune inflammatory index (SII) values (*p* < 0.001). Additionally, the prevalence of sarcopenia among participants significantly decreased from Q1 to Q4 (*p* < 0.001, Supplementary Table S1), with notably lower rates in Q4 (7.49%) than in Q1 (10.28%). These variations suggest that the potential link between DI-GM and sarcopenia requires further investigation.

**Table 1:** Basic characteristics of participants according to sarcopenia status^a^.

Variable	Total (*n* = 8,872)	Non-sarcopenia (*n* = 8,099)	Sarcopenia (*n* = 773)	*p-*value
Age, mean (SE), year	39.52 (0.10)	39.12 (0.10)	43.73 (0.30)	<0.001
BMI, mean (SE), kg/m^2^	28.97 (0.06)	28.43 (0.06)	34.71 (0.22)	<0.001
PIR, mean (SE)	2.56 (0.01)	2.60 (0.01)	2.14 (0.05)	<0.001
DI-GM, mean (SE)	4.97 (0.01)	4.99 (0.01)	4.76 (0.04)	<0.001
Daily energy intake, mean (SE), (kcal/day)	2126.70 (6.12)	2145.92 (6.32)	1922.53 (17.80)	<0.001
DII, mean (SE)	0.79 (0.01)	0.76 (0.01)	1.11 (0.05)	<0.001
SII, mean (SE)	503.03 (2.95)	497.65 (2.81)	560.11 (9.22)	<0.001
Gender, *n* (%)				0.073
Female	4,591 (52.03)	4,197 (52.22)	394 (49.96)	
Male	4,281 (47.97)	3,902 (47.78)	379 (50.04)	
Race, *n* (%)				<0.001
Mexican American	1,269 (14.53)	1,006 (12.68)	263 (34.13)	
Other Hispanic	902 (10.43)	782 (9.89)	120 (16.19)	
Non-Hispanic White	3,170 (35.34)	2,952 (36.06)	218 (27.70)	
Non-Hispanic Black	1,929 (21.56)	1,876 (22.96)	53 (6.75)	
Other race—including multi-racial	1,602 (18.14)	1,483 (18.41)	119 (15.23)	
Marital status, *n* (%)				<0.001
Married	4,356 (49.42)	3,950 (49.04)	406 (53.45)	
Widowed	120 (1.34)	110 (1.38)	10 (0.95)	
Divorced	806 (8.74)	722 (8.64)	84 (9.85)	
Separated	311 (3.54)	272 (3.44)	39 (4.54)	
Never married	2,306 (26.04)	2,152 (26.58)	154 (20.30)	
Living with a partner	973 (10.92)	893 (10.92)	80 (10.91)	
Education level, *n* (%)				<0.001
Less than high school	1,484 (16.76)	1,257 (15.49)	227 (30.28)	
High school or equivalent	1,911 (21.33)	1,706 (20.99)	205 (24.94)	
College or above	5,477 (61.91)	5,136 (63.52)	341 (44.79)	
Smoking status, *n* (%)				0.211
No	5,449 (61.28)	4,959 (61.14)	490 (62.73)	
Yes	3,423 (38.72)	3,140 (38.86)	283 (37.27)	
Drinking status, *n* (%)				<0.001
No	2,227 (25.00)	1,967 (24.29)	260 (32.50)	
Yes	6,645 (75.00)	6,132 (75.71)	513 (67.50)	
Physical activity, *n* (%)				<0.001
Low	2,711 (30.89)	2,381 (29.87)	330 (41.79)	
High	6,161 (69.11)	5,718 (70.13)	443 (58.21)	
CVD, *n* (%)				<0.001
No	8,525 (96.16)	7,818 (96.59)	707 (91.59)	
Yes	347 (3.84)	281 (3.41)	66 (8.41)	
Diabetes, *n* (%)				<0.001
No	7,872 (88.35)	7,288 (89.61)	584 (74.91)	
Yes	1,000 (11.65)	811 (10.39)	189 (25.09)	
Hypertension, *n* (%)				<0.001
No	6,385 (71.88)	5,933 (73.14)	452 (58.50)	
Yes	2,487 (28.12)	2,166 (26.86)	321 (41.50)	

BMI, body mass index; PIR, poverty impact ratio; DI-GM, dietary index for gut microbiota; DII, dietary inflammatory index; SII, systemic immune inflammatory index; CVD, cardiovascular disease.

a Percentage estimates are nationally representative using survey weights.

### Association between DI-GM and sarcopenia

The correlation between the DI-GM score and sarcopenia risk is shown in Table 2. Logistic regression analysis revealed a significant negative association between DI-GM scores and the risk of sarcopenia. When DI-GM was used as a continuous variable, the odds ratio (OR) of Model 1 (M1) was 0.92 (95% CI = 0.89–0.95, *p* < 0.001). After adjusting for demographic factors (M2) and in the fully adjusted model (M3), the OR values remained significant (M2: OR = 0.90, 95% CI = 0.87–0.94, *p* < 0.001; M3: OR = 0.91, 95% CI = 0.88–0.95, *p* < 0.001). Further analysis based on the DI-GM score groupings supported these findings. The results indicated that the highest group (Q4) was significantly associated with a reduced risk of sarcopenia compared with the lowest group (Q1) across all three models (M1: OR = 0.71, 95% CI = 0.61–0.82, *p* < 0.001; M2: OR = 0.63, 95% CI = 0.53–0.75, *p* < 0.001; and M3: OR = 0.67, 95% CI = 0.56–0.80, *p* < 0.001). Moreover, trend analyses across all models demonstrated statistical significance (*p* < 0.001), further supporting the strong association between higher DI-GM scores and a decreased risk of sarcopenia.

**Table 2:** Association between DI-GM and sarcopenia.

Variables	Model 1		Model 2		Model 3	
	OR (95% CI)	*p-*value	OR (95% CI)	*p-*value	OR (95% CI)	*p-*value
DI-GM	0.92 (0.89–0.95)	<0.001	0.90 (0.87–0.94)	<0.001	0.91 (0.88–0.95)	<0.001
DI-GM group						
Q1 (0–3)	1.00 (Reference)		1.00 (Reference)		1.00 (Reference)	
Q2 (4)	0.81 (0.67–0.98)	0.033	0.70 (0.57–0.85)	<0.001	0.71 (0.58–0.87)	0.002
Q3 (5)	0.87 (0.74–1.02)	0.081	0.75 (0.63–0.88)	0.001	0.75 (0.63–0.89)	0.003
Q4 (≥6)	0.71 (0.61–0.82)	<0.001	0.63 (0.53–0.75)	<0.001	0.67 (0.56–0.80)	<0.001
*p* for trend	<0.001		<0.001		<0.001	

Model 1: Crude.

Model 2: Adjusted for sex, age, ethnicity, education level, PIR, and marital status.

Model 3: Further adjusted for smoking, drinking, CVD, diabetes, hypertension, and physical activity.

### Non-linear relationship between DI-GM and sarcopenia

**Figure 2:**
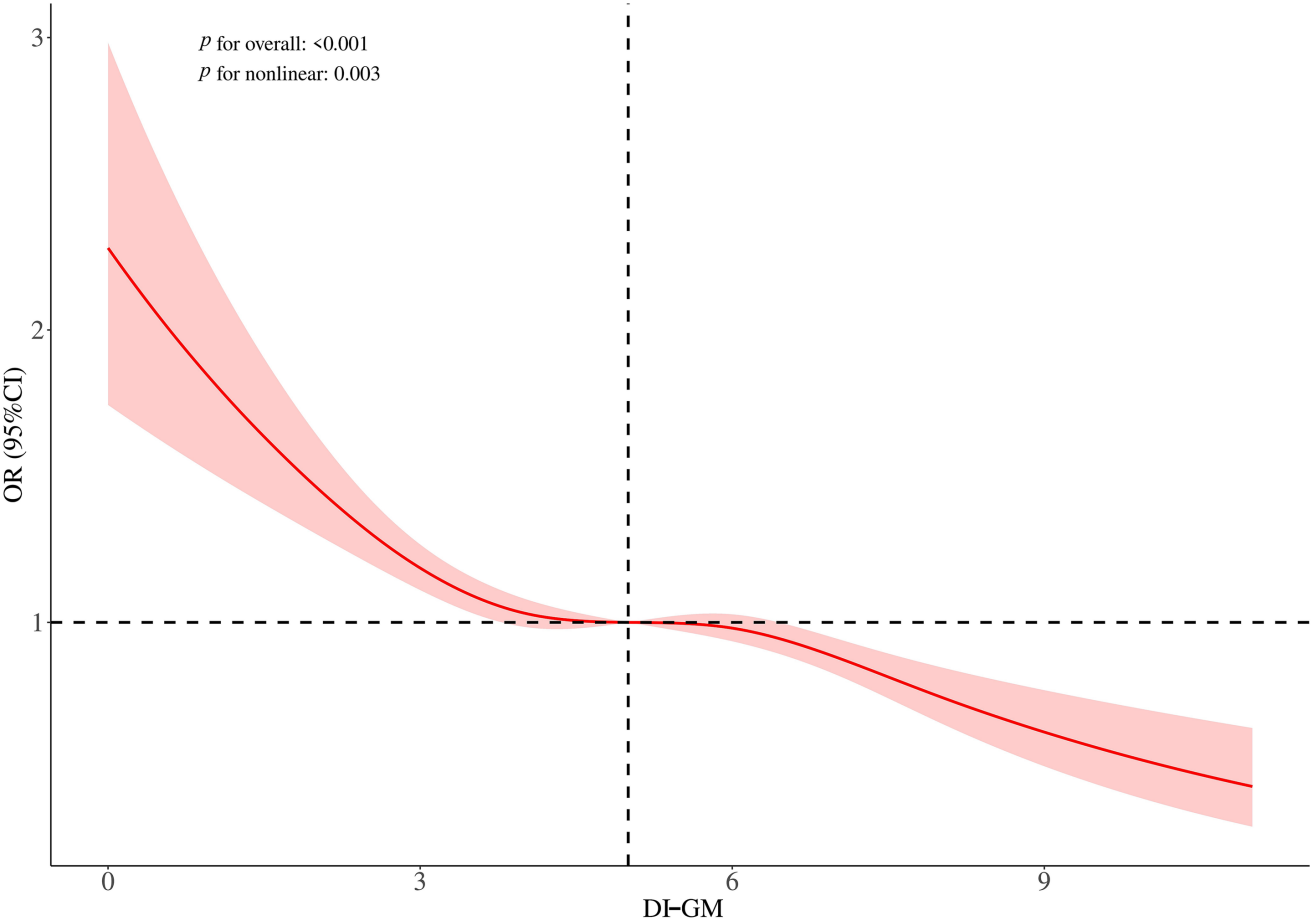
Restricted cubic spline plots for the association between DI-GM and sarcopenia. Adjusted for sex, age, ethnicity, education level, PIR, marital status, smoking, drinking, CVD, diabetes, hypertension, and physical activity.

RCS analysis was conducted to explore the relationship between DI-GM scores and the risk of sarcopenia. The findings indicated a non-linear relationship between DI-GM and the risk of sarcopenia (Figure 2). To examine this relationship in greater detail, a weighted two-segment linear regression model and a recursive algorithm were employed to conduct a threshold effect analysis. The analysis identified an inflection point at a DI-GM score of 4, with a log-likelihood ratio test showing significance at *p* = 0.008. For DI-GM scores below the threshold, each unit increase in the DI-GM was associated with a 13% reduction in the risk of sarcopenia (OR = 0.87, 95% CI = 0.77–0.98, *p* = 0.021; Table 3). However, when the DI-GM scores surpassed this threshold, each incremental unit was associated with a 6% reduction in the risk of sarcopenia (OR = 0.94, 95% CI = 0.91–0.97, *p* < 0.001; Table 3).

**Table 3:** Threshold effect analysis of DI-GM and sarcopenia^a^.

Outcome	Effect OR (95% CI)	*p-*value
DI-GM		
Model 1	0.90 (0.88–0.92)	<0.001
Model 2		
Inflection point	4	
<4	0.87 (0.77–0.98)	0.021
≥4	0.94 (0.91–0.97)	<0.001
*p* for likelihood test		0.008

Model 1: Fitting model using standard linear regression.

Model 2: Fitting model using two-piecewise linear regression.

a Adjusted for sex, age, ethnicity, education level, PIR, marital status, smoking, drinking, CVD, diabetes, hypertension, and physical activity.

### Subgroup analysis

**Figure 3:**
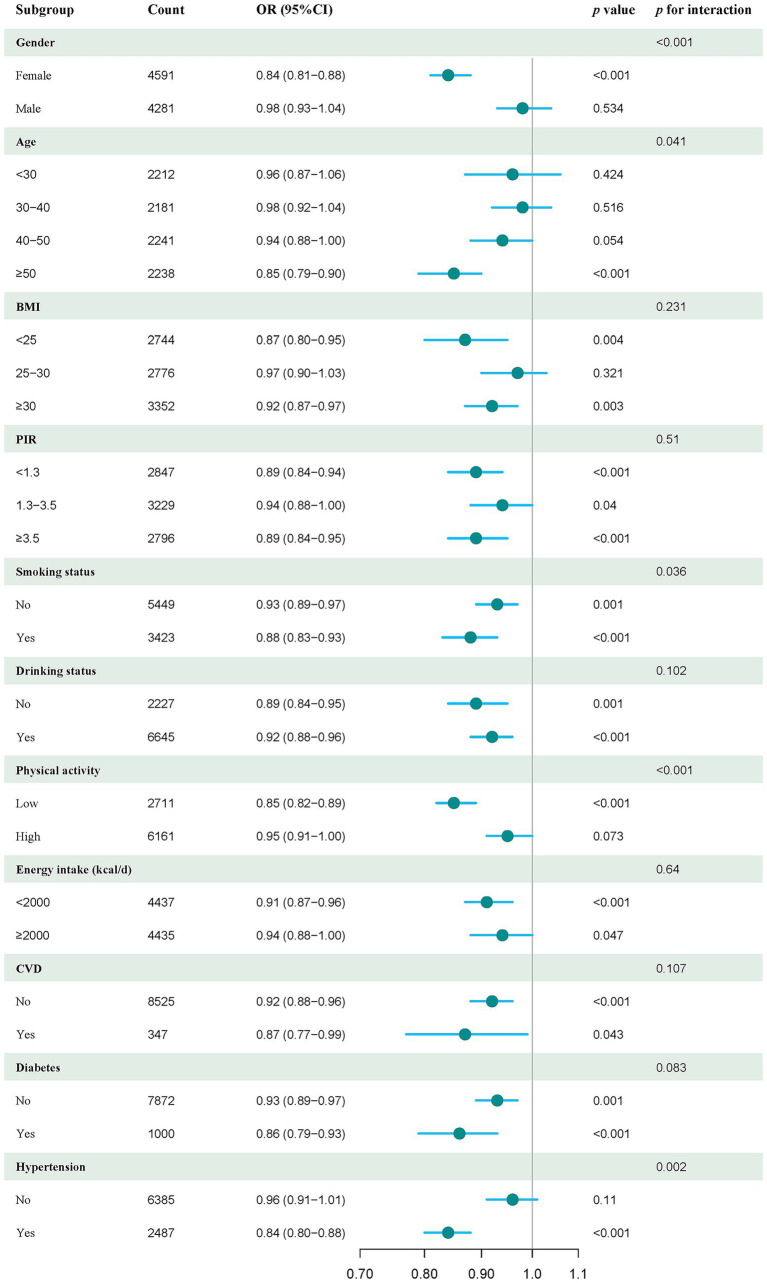
Forest plot of stratified analysis and interaction effects on the association between DI-GM and sarcopenia. The model was adjusted for sex, age, ethnicity, education level, PIR, marital status, smoking, alcohol consumption, CVD, diabetes, hypertension, and physical activity.

To further explore the link between DI-GM scores and sarcopenia risk, we examined various subgroups categorized by demographic and health factors. The findings revealed a significant inverse relationship between DI-GM and sarcopenia risk, which was consistently observed across different subgroups. These subgroups included women, individuals aged ≥50 years, those with a BMI <25 and BMI ≥30, individuals with lower levels of physical activity and daily energy intake, and those with hypertension. A notably negative association between DI-GM and sarcopenia risk was also evident in each subgroup of PIR, smoking, alcohol consumption, CVD, and diabetes (Figure 3). Additionally, we identified statistically significant interactions between DI-GM and covariates such as sex, age, smoking status, physical activity, and hypertension (*p* < 0.05, Figure 3).

### Mediation analysis

**Figure 4:**
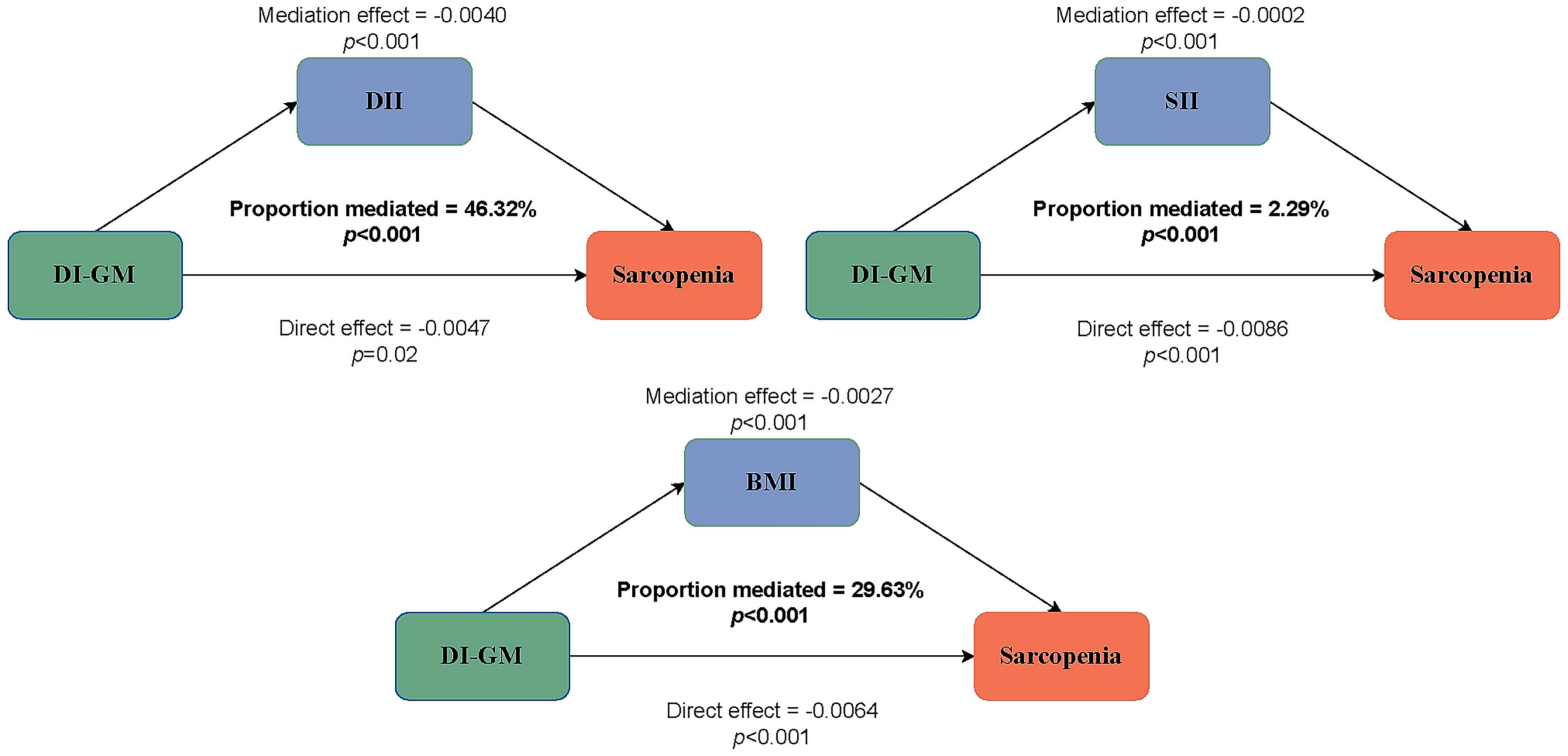
Estimated proportion of the association between DI-GM and sarcopenia mediated by DII, SII, and BMI.

To disclose the potential mediating variables in the association between DI-GM and sarcopenia, we conducted a mediation analysis to explore the mediating roles of the DII, SII, and BMI. The results showed that the mediating effects of DII, SII, and BMI on the association between DI-GM and sarcopenia in the fully adjusted model were −0.004 (*p* < 0.001), −0.0002 (*p* < 0.001), and −0.0027 (*p* < 0.001), respectively, with mediation proportions of 46.32, 2.29, and 29.63%, respectively (Figure 4).

## Discussion

In the present study, we identified an inverse relationship between DI-GM and sarcopenia risk. Elevated DI-GM scores were significantly correlated with a reduced risk of sarcopenia in older adults. Our findings indicated that individuals in the non-sarcopenia group exhibited substantially higher DI-GM scores than those in the sarcopenia group. Through RCS analysis, we discerned a non-linear association between DI-GM scores and sarcopenia risk, with enhanced protective roles observed when the DI-GM score was below the inflection point of four. Subgroup analysis revealed that DI-GM conferred protection in subgroups aged ≥50 years, with BMI <25 and BMI ≥30, lower physical activity, lower daily energy intake, and hypertension. The mediation analysis results indicated that the total DII, SII, and BMI accounted for 78.24% of the association between DI-GM and sarcopenia.

Recent studies suggest that dysbiosis of the gut microbiota may be a potential pathogenic factor in sarcopenia development (42–44). Wang et al. (45) found a notable decrease in phylum *Bacteroides* in the gut microbiota of elderly Chinese women with sarcopenia, whereas genus *Prevotella* was more abundant. Liu et al. (46) showed that mice treated with antibiotics and given fecal microbiota transplantation from elderly individuals with sarcopenia had reduced muscle mass and strength compared to those receiving microbiota from non-sarcopenic donors. However, the administration *of Lacticaseibacillus rhamnosus* and *Faecalibacterium prausnitzii* improved muscle mass and function in aged mice. Furthermore, healthy gut microbiota may reduce the risk of sarcopenia by influencing muscle function through the modulation of protein, energy, lipid, and glucose metabolism, and may even affect inflammation, neuromuscular junctions, and mitochondrial function (42, 43, 47).

Evidence has shown that dietary habits play a significant role in shaping the gut microbiota composition, underscoring the importance of nutritional indices in understanding the link between the gut microbiome and disease risk (26, 27). A healthy gut microbiome is characterized by considerable microbial richness and diversity. The gut microbiota influences the host’s dietary response, and the host can also alter the gut microbiota through changes in dietary habits. Fat- and sugar-rich diets can lead to decreased microbial diversity, lower production of metabolites crucial for maintaining gut permeability, damage to the mucus layer, increased bacterial translocation, and higher levels of lipopolysaccharide. These alterations can result in endotoxemia, chronic subclinical inflammation, and metabolic disorders (48, 49). Intestinal microbiota health is closely linked to sarcopenia in older adults (42, 43). Therefore, assessing gut microbiota health by examining dietary habits in the aging population with sarcopenia is vital for improving public health.

The DI-GM is a new dietary pattern index designed to predict gut microbiota health by identifying 14 nutritional components that can have beneficial or unfavorable effects on the gut microbiome (28). This study examined the relationship between DI-GM and the risk of sarcopenia. We found that DI-GM had a non-linear inverse association with the risk of sarcopenia. Our findings suggest that elevated DI-GM scores may protect against sarcopenia in older adult females. Furthermore, we observed that individuals with lower DI-GM scores had higher DII and SII levels, suggesting that inflammation may mediate the effect of DI-GM on sarcopenia. The results of the mediation analysis demonstrated that the DII and SII accounted for 46.32 and 2.29% of the mediated proportion, respectively, in the association between DI-GM and sarcopenia risk. These findings align with those of previous studies, suggesting that a higher dietary inflammatory index is associated with an increased risk of sarcopenia (50). This also suggests that consuming foods beneficial to gut health may help reduce inflammatory levels in the gut and immune system. Additionally, we found that BMI mediated 29.63% of the correlation between DI-GM and sarcopenia risk, supporting the protective role of DI-GM in individuals with BMI <25 and BMI ≥30. This finding aligns with prior studies indicating that both low and high BMI are associated with an increased risk of sarcopenia in older adult women (51, 52). Notably, although SII can assess local systemic inflammation and the body’s immunological response, its relatively low mediation proportion suggests that further exploration of other inflammatory markers such as C-reactive protein (CRP), interleukin-6 (IL-6), and tumor necrosis factor alpha (TNFα) is necessary.

Subgroup analysis showed that the protective roles of DI-GM in sarcopenia were consistent across various subgroups, and statistically significant interactions between DI-GM and covariates were identified in subgroups stratified by sex, age, smoking status, physical activity, and hypertension. These findings imply that these covariates contribute to variations in the correlation between DI-GM and sarcopenia in different subgroups. Notably, hormonal differences may account for the interactions between DI-GM and adult females regarding sarcopenia risk. Similarly, individuals aged ≥50 years, smokers, those with lower physical activity, and those with hypertension tend to have a higher risk of sarcopenia due to the modulatory effects of aging, inflammation, muscle function, energy, and metabolic disorders (42, 43, 47).

The association between higher DI-GM scores and a decreased risk of sarcopenia highlights the potential effectiveness of dietary interventions in improving gastrointestinal health. Notably, our threshold effect analysis revealed a more pronounced protective association when the DI-GM score was below the inflection point of four. According to the DI-GM calculation methodology, an increase in the score corresponds to a decrease in the consumption of dietary components with high-energy densities, such as red meat and high-fat milk (28). Our results suggest that adequate protein intake substantially protects against sarcopenia, as demonstrated by DI-GM scores of <4. These findings suggest that the optimal strategy for older adults involves balancing protein intake and beneficial ingredients for gut health, as represented by the DI-GM, with less-favorable ingredients. This equilibrium is crucial for muscle health because it regulates energy and nutrition.

This study had several notable strengths. After adjusting for confounding variables, this is the first study to demonstrate a significant association between DI-GM and sarcopenia mediated by inflammaging and BMI. These findings suggest that a higher DI-GM score is correlated with a decreased risk of sarcopenia in older women. Furthermore, this study identified a non-linear relationship between DI-GM and the risk of sarcopenia. Subgroup analyses further substantiated the robustness of these results. Future longitudinal studies should investigate the combined effects of dietary and gut microbiota interventions on muscle health across diverse populations to validate these results.

Our study had several limitations. First, the cross-sectional design limits our capacity to establish a causal relationship between DI-GM and the risk of sarcopenia, highlighting the necessity for longitudinal and prospective studies. Second, the DI-GM scores were calculated based on the intake data from 14 food components, resulting in participant exclusion when data were missing, which may have introduced selection bias. Moreover, the binary scoring of DI-GM, which relies on median dichotomization, may oversimplify the intricacies of dietary patterns, leading to a loss of detailed information. Additionally, reliance on self-reported dietary information from two 24-h dietary recall interviews increased the potential for recall bias. Third, despite adjustments for numerous potential confounders, the possibility of residual confounding and unmeasured factors, such as dietary supplement use and undiagnosed muscular disorders, cannot be completely ruled out. Fourth, the DI-GM reflects dietary habits during data collection rather than long-term patterns; however, most adults maintain consistent diets unless they experience significant health issues, suggesting that the DI-GM reasonably represents typical dietary habits. Fourth, sarcopenia is influenced by lifestyle factors such as occupational stress, inadequate sleep, and sedentariness. However, the DI-GM score does not comprehensively capture the influence of these factors on the risk of sarcopenia. Finally, the generalizability of the study findings is limited because significant associations were primarily observed in the U.S. population. A more diverse population is necessary to validate these associations and enhance our understanding of the relationship between diet, gut microbiota, and the risk of sarcopenia. Future research should consider longitudinal or interventional study designs and microbiome sequencing data to determine the causal relationship between DI-GM and sarcopenia.

## Conclusion

This study found a significant negative association between DI-GM and sarcopenia risk. Interestingly, the relationship between the DI-GM scores and sarcopenia risk demonstrated a non-linear pattern and was partially mediated by inflammaging and BMI. As a new dietary quality index that reflects gut microbiota diversity, further longitudinal or interventional studies using DI-GM could help to develop strategies to prevent and reduce sarcopenia risk.

## Data Availability

The datasets presented in this study can be found in online repositories. The names of the repository/repositories and accession number(s) can be found at: https://wwwn.cdc.gov/nchs/nhanes/Default.aspx.

## Footnotes

^1^ https://www.cdc.gov/nchs/nhanes/index.htm
