# Supplementary material for: The dietary inflammation index and body mass index mediate the association between the dietary index for gut microbiota and sarcopenia in older women: evidence from NHANES 2011–2018
Source: Front Nutr. 2025 Aug 18;12:1624844. doi: 10.3389/fnut.2025.1624844 (PMC12399385; doi:10.3389/fnut.2025.1624844)
Supplement: Supplementary file 1 [file Table_1.docx]

| **Table S1. Basic characteristics of the study population based on DI-GM scores*.** | | | | | | |
| --- | --- | --- | --- | --- | --- | --- |
| **Variable** | **Total**  **(n = 8872)** | **Q1**  **(0-3, n=1675)** | **Q2**  **(4, n=1890)** | **Q3**  **(5, n=2141)** | **Q4**  **(≥6, n=3166)** | ***p* value** |
|  |  |  |  |  |  |  |
| **Age, mean (SE), year** | 39.52 (0.10) | 38.75 (0.21) | 39.19 (0.19) | 39.08 (0.18) | 40.43 (0.13) | <0.001 |
| **BMI, mean (SE), kg/m^2^** | 28.97 (0.06) | 29.90 (0.12) | 29.18 (0.11) | 29.07 (0.10) | 28.27 (0.08) | <0.001 |
| **PIR, mean (SE)** | 2.56 (0.01) | 2.26 (0.03) | 2.38 (0.03) | 2.47 (0.03) | 2.88 (0.02) | <0.001 |
| **Daily energy intake,**  **mean (SE), (kcal/d)** | 2126.70  (6.12) | 2046.08 (14.41) | 2075.49  (13.43) | 2136.31 (12.61) | 2193.63  (9.08) | <0.001 |
| **DII, mean (SE)** | 0.79 (0.01) | 1.53 (0.02) | 1.24 (0.03) | 0.86 (0.03) | 0.07 (0.02) | <0.001 |
| **SII, mean (SE)** | 503.03 (2.95) | 511.23 (6.65) | 504.75 (4.49) | 504.20 (5.75) | 496.82 (3.91) | 0.040 |
| **Gender, n (%)** |  |  |  |  |  | <0.001 |
| Female | 4591 (52.03) | 815 (49.63) | 931 (49.06) | 1139 (53.18) | 1706 (54.28) |  |
| Male | 4281 (47.97) | 860 (50.37) | 959 (50.94) | 1002 (46.82) | 1460 (45.72) |  |
| **Race, n (%)** |  |  |  |  |  | <0.001 |
| Mexican American | 1269 (14.53) | 215 (12.72) | 286 (15.70) | 351 (16.84) | 417 (13.17) |  |
| Other Hispanic | 902 (10.43) | 170 (10.55) | 203 (10.47) | 216 (10.22) | 313 (10.49) |  |
| Non-Hispanic White | 3170 (35.34) | 554(32.87) | 664 (34.22) | 767 (35.71) | 1185 (37.06) |  |
| Non-Hispanic Black | 1929 (21.56) | 497 (29.25) | 462 (24.82) | 453 (20.67) | 517 (16.15) |  |
| Other Race - Including  Multi-Racial | 1602 (18.14) | 239 (14.62) | 275 (14.78) | 354 (16.57) | 734 (23.12) |  |
| **Marital status, n (%)** |  |  |  |  |  | <0.001 |
| Married | 4356 (49.42) | 736(44.90) | 872 (47.01) | 1070 (49.50) | 1678 (53.22) |  |
| Widowed | 120 (1.34) | 32 (2.01) | 27 (1.24) | 22 (1.13) | 39 (1.20) |  |
| Divorced | 806 (8.74) | 155 (9.09) | 177 (9.30) | 201 (8.79) | 273 (8.19) |  |
| Separated | 311 (3.54) | 57 (3.29) | 70 (3.68) | 70 (3.27) | 114 (3.77) |  |
| Never married | 2306 (26.04) | 483 (27.63) | 530 (27.56) | 548 (26.48) | 745 (23.97) |  |
| Living with partner | 973 (10.92) | 212 (13.09) | 214 (11.21) | 230 (10.83) | 317 (9.65) |  |
| **Education level, n (%)** |  |  |  |  |  | <0.001 |
| Less than high school | 1484 (16.76) | 295 (17.65) | 386 (20.45) | 397 (19.02) | 406 (12.48) |  |
| High school or equivalent | 1911 (21.33) | 463 (27.77) | 470 (25.69) | 469 (21.43) | 509 (15.21) |  |
| College or above | 5477 (61.91) | 917 (54.58) | 1034 (53.86) | 1275 (59.55) | 2251 (72.31) |  |
| **Smoking status, n (%)** |  |  |  |  |  | <0.001 |
| No | 5449 (61.28) | 983 (58.97) | 1123 (59.35) | 1300 (60.72) | 2043 (64.06) |  |
| Yes | 3423 (38.72) | 692 (41.03) | 767 (40.65) | 841 (39.28) | 1123 (35.94) |  |
| **Drinking status, n (%)** |  |  |  |  |  | 0.017 |
| No | 2227 (25.00) | 412 (25.32) | 494 (26.56) | 538 (24.99) | 783 (23.89) |  |
| Yes | 6645 (75.00) | 1263 (74.68) | 1396 (73.44) | 1603 (75.01) | 2383 (76.11) |  |
| **Physical activity, n (%)** |  |  |  |  |  | <0.001 |
| Low | 2711 (30.89) | 548 (33.15) | 599 (32.37) | 631 (29.83) | 933 (29.56) |  |
| High | 6161 (69.11) | 1127 (66.85) | 1291 (67.63) | 1510 (70.17) | 2233 (70.44) |  |
| **CVD, n (%)** |  |  |  |  |  | <0.001 |
| No | 8525 (96.16) | 1598 (95.56) | 1806 (95.72) | 2049 (95.69) | 3072 (97.07) |  |
| Yes | 347 (3.84) | 77 (4.44) | 84 (4.28) | 92 (4.31) | 94 (2.93) |  |
| **Diabetes, n (%)** |  |  |  |  |  | <0.001 |
| No | 7872 (88.35) | 1451 (86.14) | 1668 (88.01) | 1890 (88.15) | 2863 (89.87) |  |
| Yes | 1000 (11.65) | 224 (13.86) | 222 (11.99) | 251 (11.85) | 303 (10.13) |  |
| **Hypertension, n (%)** |  |  |  |  |  | <0.001 |
| No | 6385 (71.88) | 1166 (68.84) | 1332 (71.15) | 1535 (72.09) | 2352 (73.80) |  |
| Yes | 2487 (28.12) | 509 (31.16) | 558 (28.85) | 606 (27.91) | 814 (26.20) |  |
| **Sarcopenia, n (%)** |  |  |  |  |  | <0.001 |
| No | 8099 (91.39) | 1498 (89.72) | 1725 (91.51) | 1949 (90.98) | 2927 (92.51) |  |
| Yes | 773 (8.61) | 177 (10.28) | 165 (8.49) | 192 (9.02) | 239 (7.49) |  |
| BMI, body mass index; PIR, poverty impact ratio; DI-GM, dietary index for gut microbiota; DII, dietary inflammatory index; SII, systemic immune inflammatory index; CVD, cardiovascular disease.  *Percentage estimates are nationally representative using survey weights. | | | | | | |
